# Differences in clinical characteristics and chest CT findings between severe and critical H1N1 pneumonia

**DOI:** 10.1111/crj.13591

**Published:** 2023-02-01

**Authors:** Mei Yu, Yuanbo Zhu, Xiaoyan Qu, Xingyi Hou, Tao Xin, Gangfeng Li

**Affiliations:** ^1^ Department of Radiology Tangdu Hospital, Air Force Medical University Xi'an People's Republic of China; ^2^ Department of Respiratory and Critical Care Medicine Tangdu Hospital, Air Force Medical University Xi'an People's Republic of China

**Keywords:** computed tomography, critical, H1N1, pneumonia

## Abstract

**Introduction:**

Critical H1N1 pneumonia patients usually have one of the symptoms such as respiratory failure, septic shock, multiple organ dysfunction, or other need for intensive care management, which are associated with high risk of mortality. It is essential to differentiate the severity of H1N1 pneumonia and take corresponding target treatments.

**Objectives:**

We aim to investigate the differences in clinical characteristics and chest computed tomography (CT) findings between severe and critical patients with H1N1 pneumonia.

**Methods:**

A total of 27 patients diagnosed with H1N1 pneumonia from October 2018 to March 2019 were retrospectively analyzed, and the differences in clinical manifestations, laboratory tests, and chest CT findings between the severe group (15 patients) and the critical group (12 patients) were compared.

**Results:**

Frequency of dyspnea at rest was higher in critical group than that in severe group (*P* = 0.019). The neutrophil percentage was higher (*P* = 0.014) and the lymphocyte percentage was lower (*P* = 0.025) in critical compared with severe group. Bilateral lung involvement was the predominant pattern in both severe and critical patients, whereas the number of involved lobes in critical patients was more than that in severe patients (*P* = 0.024). Peripheral distribution was the predominant pattern in severe patients (40%), whereas more diffuse involvement of the lungs was observed in critical patients (83.30%). Ground‐glass opacities and consolidation were the main CT findings in both groups, and prevalence of consolidation was higher in critical relative to severe group (83.30%).

**Conclusion:**

Compared with severe patients, those with critical H1N1 pneumonia were more likely to present with dyspnea at rest and decreased lymphocyte percentage. Chest CT showed that diffuse bilateral involvement and higher prevalence of consolidation are associated with critical outcomes.

## Graphical abstract

Compared with severe patients, those with critical H1N1 pneumonia were more likely to present with dyspnea at rest, lower lymphocyte percentage. Chest computed tomography (CT) imaging showed more diffuse bilateral involvement and a higher rate of consolidation in critical compared with severe patients. CT findings, along with the help of clinical symptoms and laboratory tests, could contribute to early identification of critical H1N1 pneumonia from those severe patients.

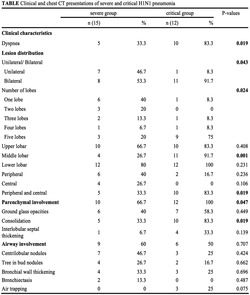

## 1 INTRODUCTION

Influenza A (H1N1) virus was first discovered at the border between Mexico and the United States in April 2009, which was historically responsible for the worldwide pandemic.^1^, ^2^ H1N1 virus infection has been deemed as seasonal influenza, resulting in substantial morbidity and hospitalizations in recent years.^3^ It broke out in certain parts of China from the end of 2018 to the beginning of 2019. Most patients with H1N1 virus infection were mild and self‐limited with good prognosis, and about 2% of the patients with H1N1 developed severe and critical diseases. Compared with severe patients with low risk of mortality, the mortality of critical patients was as high as 48.6%.^4^ Therefore, it is essential to differentiate critical patients with HINI from those severe patients and those taking precise therapy.

Previous studies^5^, ^6^, ^7^, ^8^ demonstrated that ground‐glass opacities (GGOs), consolidation, and centrilobular nodules are the most common radiological features observed in computed tomography (CT) images in patients with H1N1 pneumonia. However, little attention has been paid to compare features between severe and critical H1N1 pneumonia. In the current study, we retrospectively examined the clinical characteristics and chest CT findings of patients with H1N1 pneumonia, hoping to reveal the clinical and radiologic differences between severe and critical H1N1 pneumonia.

## 2 MATERIALS AND METHODS

### 2.1 Patient population

**FIGURE 1:**
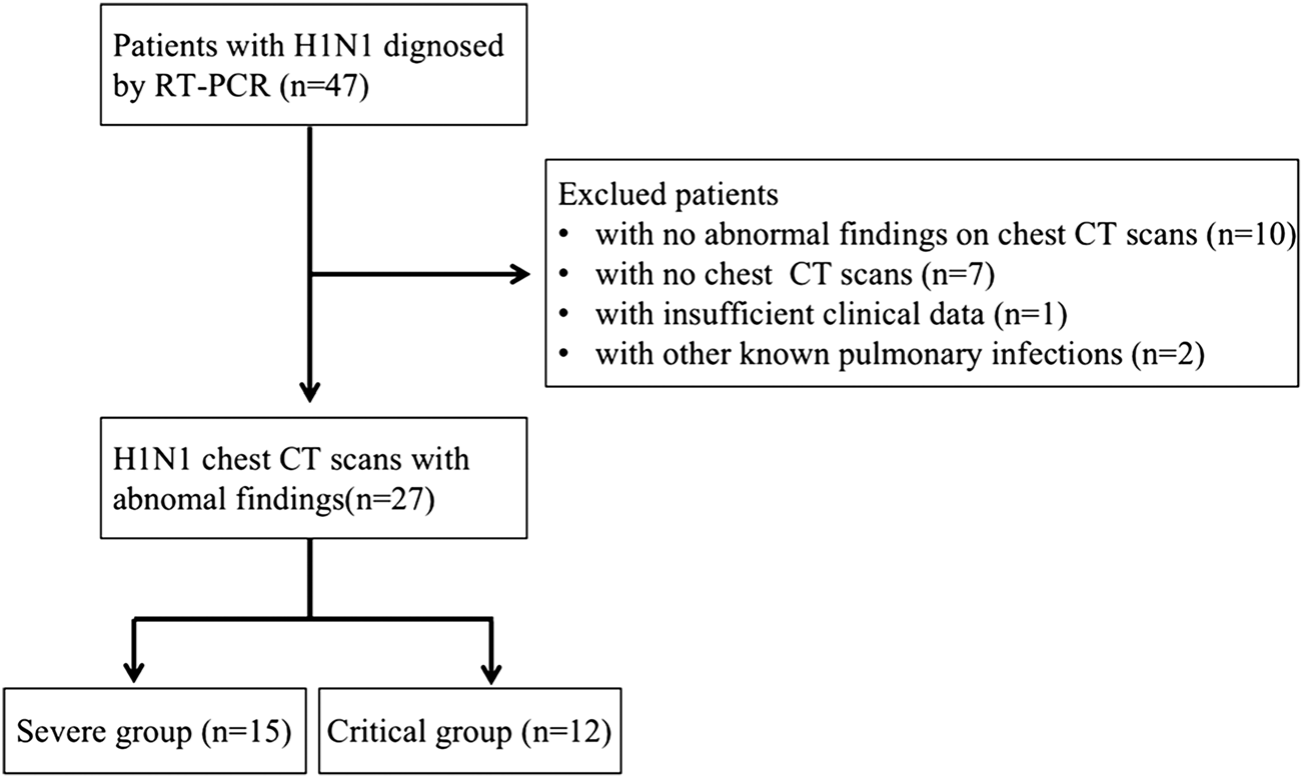
Flow diagram for the patients' inclusion and exclusion criteria.

A total of 47 patients with H1N1 virus infection from October 2018 to March 2019 were retrospectively investigated in the current study. H1N1 virus was confirmed with reverse transcription‐polymerase chain reaction (RT‐PCR) of nasal swab specimens. Exclusion criteria were as follows: (1) patients with no chest CT scans; (2) patients with no abnormal chest CT findings, namely mild patients; (3) patients with insufficient clinical data; and (4) patients with other known pulmonary infections. Finally, 27 patients were enrolled in this study and were divided into severe (15 patients) and critical (12 patients) group according to the guideline of Influenza A (H1N1)^4^ (Figure 1). The criteria were as follows:

Severe: cases who had at least one of the following symptoms: (1) high fever (>38.5°C) lasting for >3 days, accompanied with severe cough with purulent or bloody sputum, or chest pain; (2) tachypnea, dyspnea, and cyanosis; (3) dull reaction, hypersomnia, and restlessness; (4) severe vomiting, diarrhea, and dehydration; (5) pneumonia; and (6) the underlying diseases significantly aggravated. Critical: cases who had at least one of the following symptoms: (1) respiratory failure; (2) infection shock; (3) multiple organ dysfunction; and (4) other serious clinical conditions requiring intensive care management.

### 2.2 Clinical data acquisition

Demographic information, clinical manifestations and laboratory tests of all patients were retrieved from the electronic medical records, including age, gender, underlying diseases, clinical manifestations (fever, body temperature, cough, chest pain, dyspnea at rest, disorders of consciousness, and infection shock), and laboratory tests (leukocyte count, neutrophil count, lymphocyte count, neutrophil percentage, lymphocyte percentage, and C‐reactive protein).

### 2.3 CT image acquisition

CT examinations were conducted on a 64‐slice multi‐detector CT scanner (MDCT, LightSpeed VCT, GE Healthcare, Waukesha, WI, USA) using the following parameters: range from lung apex to lung bottom; tube voltage: 120 kV; tube current: 400 mA; slice thickness: 0.625 mm; image matrix: 512 × 512; pitch: 1.375:1; and rotation time: 0.4 s. All images were acquired at the end of expiration. The images were reconstructed in transverse, sagittal, and coronal planes with a slice thickness of 1.25 mm and an increment of 1.25 mm, adjusted to appropriate window levels for the lung parenchyma (width, 1500 HU; level, −500 HU) or the mediastinum (width, 400 HU; level, 40 HU), respectively.

### 2.4 CT image analysis

Two radiologists (with 13 and 9 years of experience) blinded to the clinical severity of patients independently reviewed the radiological findings. For discordant cases, final decisions were reached by consensus between the radiologists. The following CT features were evaluated:

1. Lesion distribution: bilateral/unilateral, upper/middle/lower lobe, peripheral (involving the outer one‐third of the lung) /central (involving the inner two‐thirds of the lung) /peripheral and central, number of pulmonary lobes.
2. CT signs of pulmonary parenchyma involvement, including GGOs, consolidation, and interlobular septal thickening.
3. CT signs of airway involvement, including tree in bud nodules, centrilobular nodules, bronchial wall thickening, bronchiectasis, and air trapping.
4. Extrapulmonary CT findings, including pleural effusion, pericardial effusion, and lymph nodes.
5. Dynamical changes in these aforementioned CT features.

### 2.5 Statistical analysis

The statistical analysis was performed using the IBM SPSS Statistics (version 26.0, SPSS, Chicago). The continuous variables were expressed as median and quartiles (25th and 75th percentiles). The categorical variables were estimated as number and percentages of patients. Demographic (except for age), clinical manifestations (except for body temperature), and CT findings were compared between severe and critical groups using the Fisher's exact test. Age, body temperature, and laboratory tests were compared between the two groups using the Mann–Whitney's *U*‐test. *P*‐values were adjusted for variables on CT findings by using the Benjamini–Hochberg method. *P*‐value <0.05 was considered statistically significant.

## 3 RESULTS

### 3.1 Demographic, clinical manifestations, and laboratory tests

There was no statistical difference in age, gender, body temperature, fever, cough, chest pain, shock, and disturbance of consciousness between the two groups. However, frequency of dyspnea at rest was higher in critical patients (83.30% vs. 33.33%, *P* = 0.019) than that in severe group. Patients in the critical relative to the severe group had higher neutrophil percentage (*P* = 0.014) and lower lymphocyte percentage (*P* = 0.025). There was no significant difference in the leukocyte, neutrophil, and lymphocyte count, and C‐reactive protein between the two groups (Tables 1 and 2).

**TABLE 1:** Demographic and clinical characteristics between severe and critical patients with H1N1 pneumonia.

	Severe group		Critical group		*P‐*values
	*n* (15)	%	*n* (12)	%	
Gender (male)	9	75.0	10	83.3	0.696
Age (years)^a^	62.0	44.0, 65.0	57.5	37.8, 68.3	0.867
Temperature (°C)^a^	39.5	38.9, 40.0	39.0	38.9, 40.0	0.961
Fever	13	86.7	12	100.0	0.487
Cough	11	66.7	11	91.7	0.182
Dyspnea at rest	5	33.3	10	83.3	**0.019**
Chest pain	6	40.0	7	58.3	0.449
Disorders of consciousness	0	0.0	2	16.7	0.188
Infection shock	0	0.0	1	8.3	0.444
Underlying diseases	11	73.3	11	91.7	0.342
Malignant tumor	4	26.7	1	8.3	0.342
Cardiovascular disease	2	13.3	3	25.0	0.628
Chronic kidney failure	2	13.3	0	0.0	0.487
Chronic liver disease	1	6.7	2	16.7	0.569
Diabetes	2	13.3	3	25.0	0.628
Arthritis	0	0.0	1	8.3	0.444
Pneumoconiosis	0	0.0	1	8.3	0.444

*Note*: Unless otherwise indicated, data were numbers and percentages of patients and were compared between the two groups using the Fisher's exact test.

^a^ Data were median and quartiles (25th and 75th percentiles) and were compared between the two groups using the Mann–Whitney's *U*‐test.

**TABLE 2:** Laboratory tests of severe and critical patients with H1N1 pneumonia.

	Severe group	Critical group	*P*‐value
	Median (25th, 75th)	Median (25th, 75th)	
Leukocyte count (×10^9^/L)	6.3 (5.5, 10.4)	9.8 (7.3, 20.3)	0.083
Neutrophil count (×10^9^/L)	5.1 (3.5, 8.8)	8.6 (5.6, 12.8)	0.083
Neutrophil percentage (%)	78.8 (64.2 80.5)	84.8 (74.3, 88.3)	**0.014**
Lymphocyte count (×10^9^/L)	1.1 (0.6, 1.3)	0.8 (0.3, 1.4)	0.347
Lymphocyte percentage (%)	12.7 (9.6, 22.2)	7.3 (4.9, 11.3)	**0.025**
C reaction protein	24.2 (10.7, 64.7)	44.5 (21.2, 150.7)	0.116

*Note*: Data were compared between the two groups using the Mann–Whitney's U‐test.

### 3.2 Initial chest CT presentations

Bilateral involvement was the predominant pattern in both severe and critical groups, but more involved lobes were observed in critical patients (*P* = 0.024, vs. severe patients). All lobes of bilateral lungs were involved in nine (75%) of the critical patients, and the middle lobe of the right lung was more likely to be involved in critical patients (11 patients, 91.7%) than that in severe patients (4 patients, 26.7%), whereas the upper and lower lobe involvement was not significantly different between the two groups. Peripheral distribution was the predominant pattern in severe patients (6 patients, 40%), whereas more diffuse involvement of mixed peripheral and central zones was seen in critical patients (10 patients, 83.30%). Pulmonary parenchyma involvement (mainly GGOs and consolidation) was the main CT findings in both groups. GGOs were confirmed in six (40%) of the severe patients and seven (58.3%) of the critical patients with no significant difference. The prevalence of consolidation was higher in critical group (10 patients, 83.30%) compared with that in severe group (5 patients, 33.3%). Interlobular septal thickening was noted in four critical patients and one severe patient, with no significant difference. There was no significant difference in airway involvement (tree in bud nodules, centrilobular nodules, bronchial wall thickening, bronchiectasis, and air trapping), lymph node enlargement, and pericardial effusion, between the two groups. After correction for multiple comparisons, the prevalence of the middle lobe involvement was significantly higher in critical group compared with that in severe group (adjusted *P* = 0.021); the other variables on CT findings showed no significant difference between the two groups (Table 3).

**TABLE 3:** Initial chest CT presentations of severe and critical patients with H1N1 pneumonia.

CT presentations	Severe group		Critical group		*P*‐values	Adjusted *P‐*value^a^
	*n* (15)	%	*n* (12)	%		
**Lesion distribution**						
Unilateral/bilateral					**0.043**	0.181
Unilateral	7	46.7	1	8.3		
Bilateral	8	53.3	11	91.7		
Number of lobes					**0.024**	0.126
One lobe	6	40.0	1	8.3		
Two lobes	3	20.0	0	0.0		
Three lobes	2	13.3	1	8.3		
Four lobes	1	6.7	1	8.3		
Five lobes	3	20.0	9	75.0		
Upper lobar	10	66.7	10	83.3	0.408	0.659
Middle lobar	4	26.7	11	91.7	**0.001**	**0.021**
Lower lobar	12	80.0	12	100.0	0.231	0.485
Peripheral	6	40.0	2	16.7	0.236	0.451
Central	4	26.7	0	0.0	0.106	0.278
Peripheral and central	5	33.3	10	83.3	**0.019**	0.199
**Parenchymal involvement**	10	66.7	12	100.0	**0.047**	0.165
Ground glass opacities	6	40.0	7	58.3	0.449	0.629
Consolidation	5	33.3	10	83.3	**0.019**	0.199
Interlobular septal thickening	1	6.7	4	33.3	0.139	0.324
**Airway involvement**	9	60.0	6	50.0	0.707	0.707
Centrilobular nodules	7	46.7	3	25.0	0.424	0.636
Tree in bud nodules	4	26.7	2	16.7	0.662	0.818
Bronchial wall thickening	4	33.3	3	25.0	0.696	0.769
Bronchiectasis	2	13.3	0	0.0	0.487	0.639
Air trapping	0	0.0	3	25.0	0.075	0.225
**Extrapulmonary findings**						
Pleural effusion	5	33.3	7	58.3	0.258	0.452
Pericardial effusion	4	33.3	3	25.0	0.696	0.769
Lymph node enlargement	4	26.7	2	16.7	0.662	0.818

*Note*: Data were compared between the two groups using the Fisher's exact test.

^a^ *P*‐values were adjusted for 21 variables by using the Benjamini–Hochberg method.

### 3.3 Follow‐up CT comparisons

**FIGURE 2:**
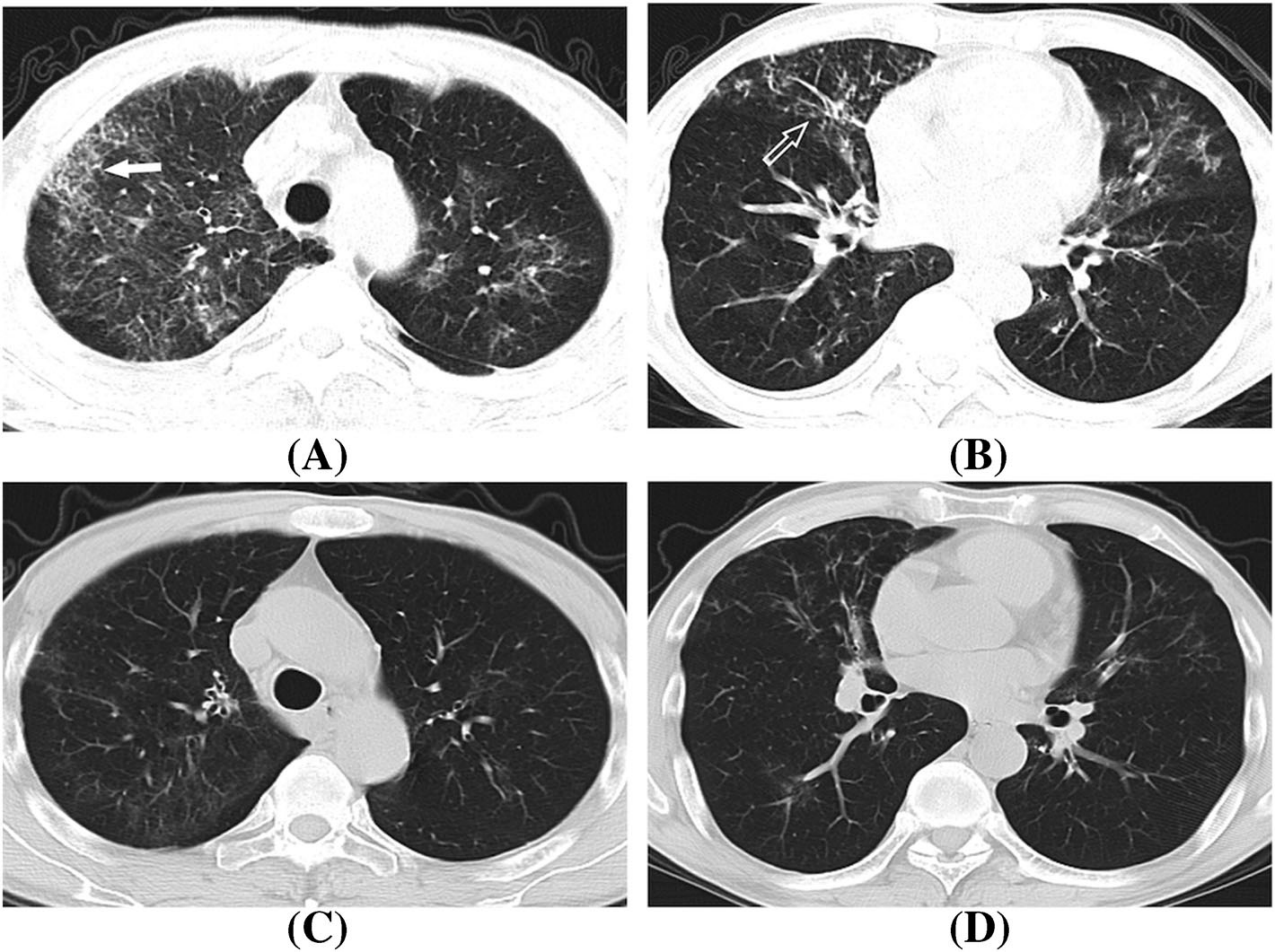
A 62‐year‐old man with severe H1N1 pneumonia, presented with fever and cough. (A, B) Axial CT showed scattered GGOs, interlobular septal thickening (white arrow), and bronchial wall thickening (white hollow arrow) in bilateral lungs. (C, D) The lesions were obviously absorbed after treatment for half a month.

**FIGURE 3:**
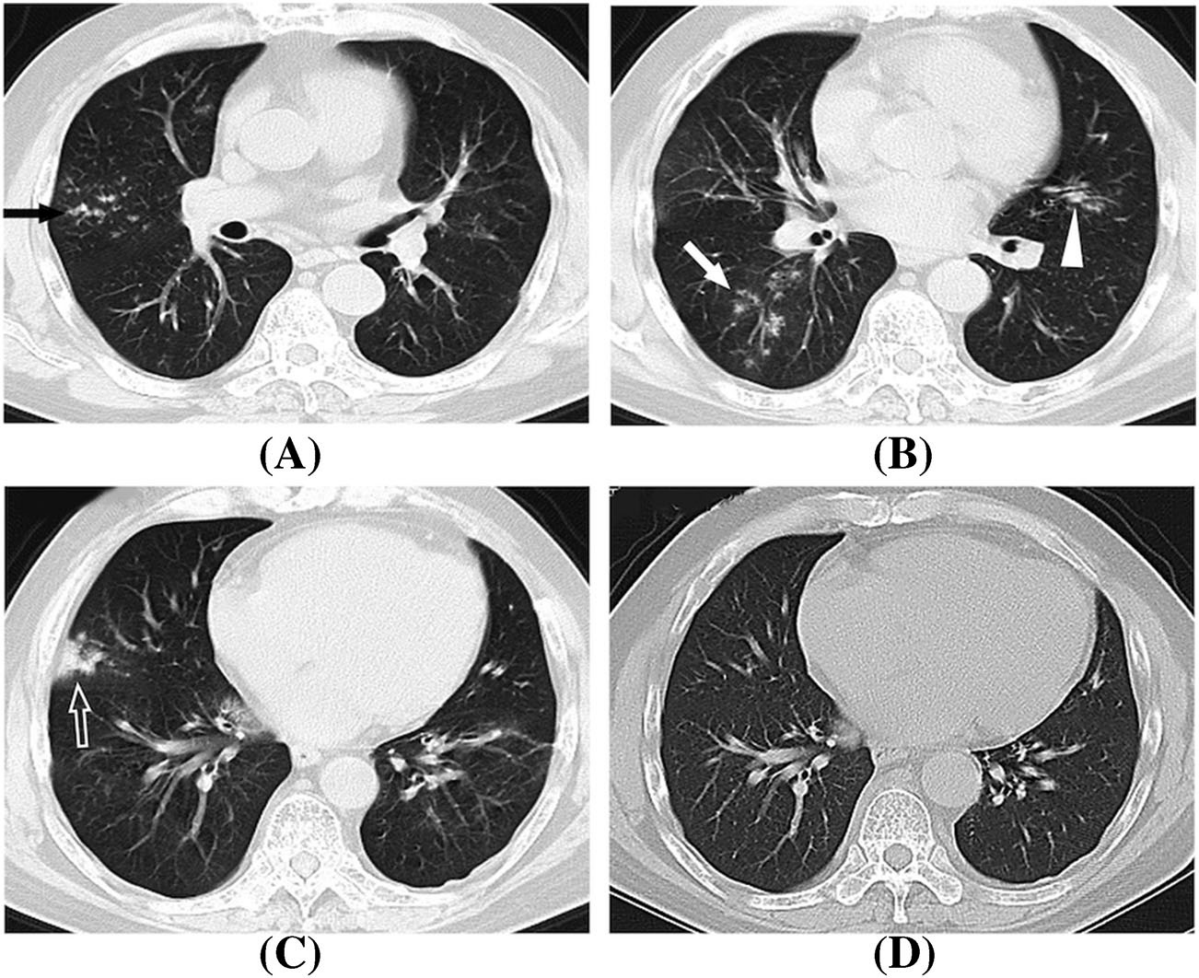
A 52‐year‐old man with severe H1N1 pneumonia, presented with fever and cough. (A–C) Axial CT showed tree in bud nodules (black arrow), centrilobular nodules (white arrow), bronchial wall thickening (white triangle), and focal consolidation (white hollow arrow) with peripheral and central distribution. (D) The lesions were completely absorbed after treatment for 8 days.

The symptoms were alleviated after anti‐viral and symptomatic treatment in all 15 severe patients with lesion shrinkage demonstrated with CT (Figure 2). Two patients (13.3%) who presented with centrilobular nodules and focal areas of consolidation at the initial chest CT showed complete lesion absorption after 1 week of treatment (Figure 3).

**FIGURE 4:**
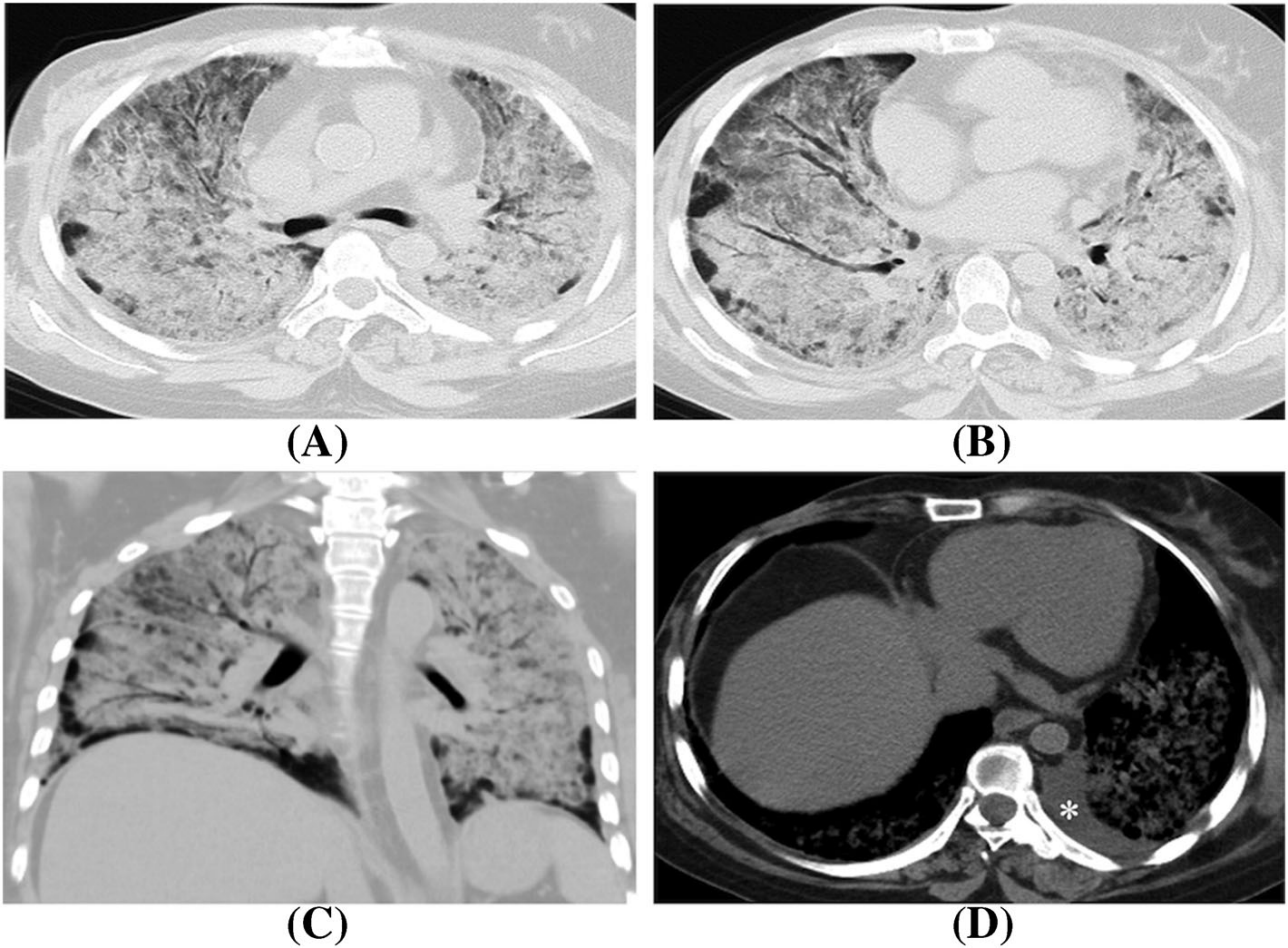
A 61‐year‐old woman with critical H1N1 pneumonia, who was admitted for sudden unconsciousness with a history of operation due to breast cancer. (A–C) Lung window showed widespread consolidation and GGOs in bilateral lungs, namely white lung. (D) Mediastinum window showed left pleural effusion (white star).

**FIGURE 5:**
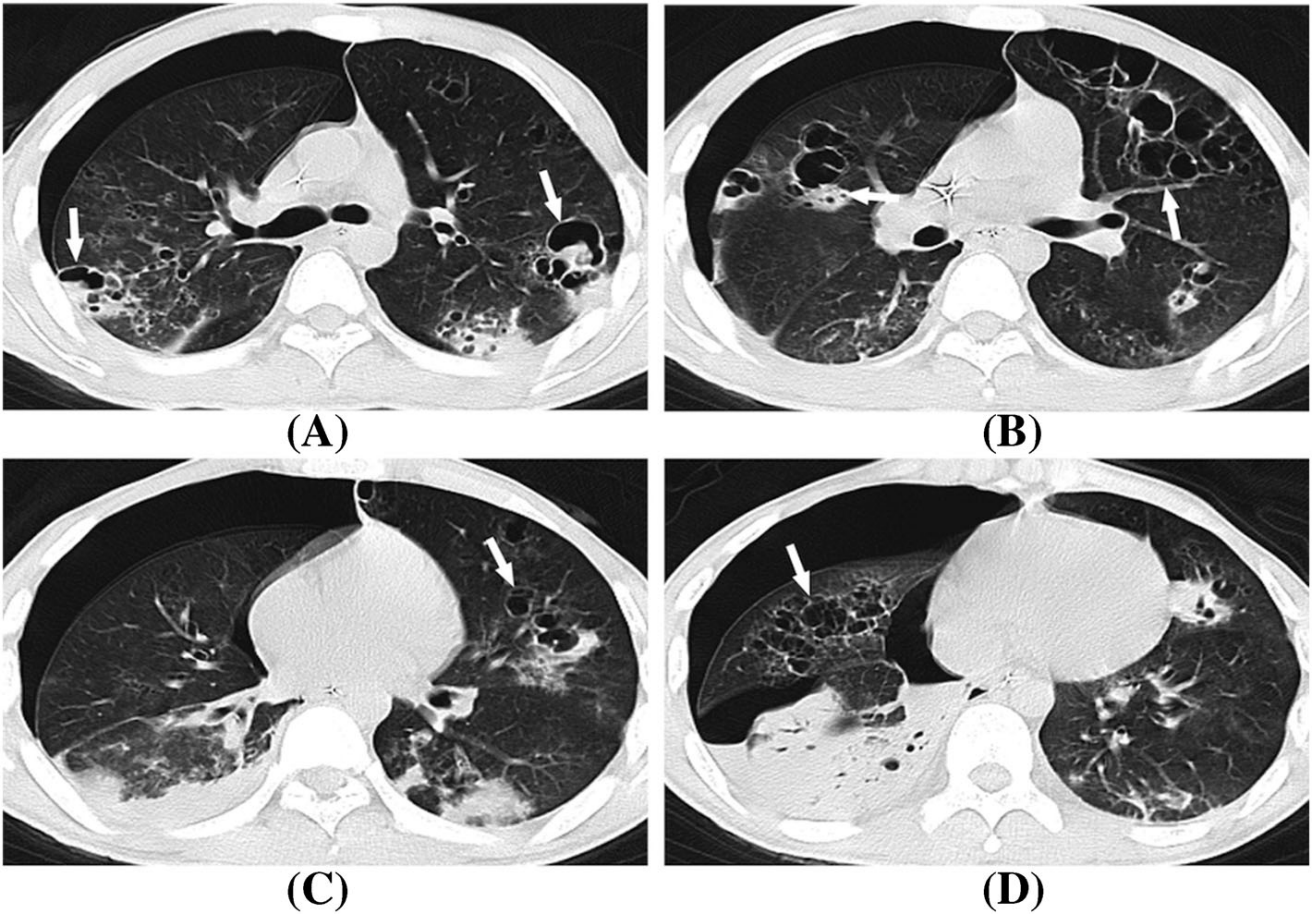
A 19‐year‐old man with critical H1N1 pneumonia, presented with fever, cough, and disturbance of consciousness. Axial CT showed multiple pneumatoceles (white arrows) in bilateral lungs and right hydropneumothorax within 17 days after the onset.

Of the 12 patients in critical group, four patients became even worse after admission, with symptoms including respiratory failure (4 patients, 33.3%), disorders of consciousness (2 patients, 16.7%), and infective shock (1 patient, 8.3%). In the follow‐up CT examination, two patients (16.7%) showed expanded lung involvement, one patient (8.3%) presented with white lung (Figure 4), and another patient (8.3%) had multiple pneumatoceles in the bilateral lung and right hydropneumothorax (Figure 5). Among the remaining eight patients (66.7%), GGOs and consolidation showed less involvement and lower density after 2–4 weeks of treatment and eventually developed to fibrosis.

## 4 DISCUSSION

In the current study, we investigated the differences in clinical characteristics and chest CT findings between severe and critical H1N1 pneumonia. Critical relative to severe group presented more frequently with dyspnea at rest (83.30% vs. 33.33%, *P* = 0.019) and lower lymphocyte percentage (*P* = 0.025). Chest CT showed more diffuse bilateral involvement and a higher rate of consolidation in critical compared with severe patients.

Previous studies^4^, ^9^, ^10^ revealed that age is an important risk factor for H1N1 pneumonia. Elder patients are prone to serious diseases because of their immunocompromised immune system and underlying diseases.^11^ Though patients included in the current study covered almost a wide age range in both groups, there was no significant difference in age and underlying diseases between the two groups. Fever, cough, chest pain, and dyspnea at rest were the major symptoms in patients with H1N1 pneumonia, and frequency of dyspnea at rest was higher in critical relative to severe patients (10 patients, 83.3%), which is consistent with one prior report.^7^ Lymphocyte percentage in critical group was lower than that in severe group (*P* = 0.025). Cheng et al.^12^ revealed that H1N1 pneumonia results in reduction in lymphocyte count, because pulmonary inflammation drives the lymphocytes to gather around the inflammation and decrease in peripheral blood. A lower lymphocyte percentage was associated with more severe outcomes.^13^, ^14^ Therefore, it is of great importance to evaluate the lymphocyte percentage in time.

The current study demonstrated that bilateral involvement was the predominant pattern in both severe and critical groups, with a slightly higher percentage in critical group (*P* = 0.043), *P* value of 0.043 was highly marginal, which cannot be accepted as being significant. Therefore, the potential clinical application value should be treated with caution. The number of involved lobes in critical patients was higher than that in severe ones (*P* = 0.024), and the middle lobe of the right lung was more likely to be involved in critical patients (11, 91.7%), even after correction for multiple comparisons (adjusted *P* = 0.021). Lee et al.^15^ observed a significantly reduced perfused blood volume (PBV) in the right middle lobe compared with all the other pulmonary lobes in patients without cardiopulmonary diseases and negative pulmonary embolism, and low‐blood‐flow regions are usually matched with low‐ventilation regions, which might influence the distribution of H1N1 pneumonia. Peripheral distribution was the predominant pattern in severe patients (6 patients, 40%), whereas more diffuse involvement of mixed peripheral and central zones of the lungs was observed in critical patients (10 patients, 83.30%), revealing an association between the scope of pulmonary inflammation and the severity of H1N1 pneumonia.

GGOs and consolidation were the main CT findings in both severe and critical patients, advocating findings of Valente et al.^16^ GGOs in the acute phase of H1N1 pneumonia may represent the inflammatory infiltrates, diffuse alveolar damage, and alveolar edema.^17^ As the disease develops, the interlobular septa is thickened due to the obstruction of lymphatic reflux.^18^ When edema is further aggravated, CT shows consolidation and even “white lung.” In the present study, the rate of consolidation was higher in critical group (10 patients, 83.30%) than that in severe group (5 patients, 33.3%). After treatment, symptoms were alleviated and size of consolidation was reduced in eight critical patients. Changes in follow‐up CT findings were along with clinical symptoms resolving. Thus, we speculate that the presence and size of consolidation are associated with the severity of outcomes.

In addition to diffuse alveolar damage,^19^ H1N1 pneumonia has necrotizing bronchiolitis.^17^, ^18^, ^20^ In this study, centrilobular nodules and tree in bud nodules corresponding to airway involvement were noted in both severe and critical patients. Air trapping was depicted in three critical patients, among whom one patient had multiple pneumatoceles in bilateral lung and right hydropneumothorax during treatment. Cristina et al.^21^ reported that the appearance of air trapping was due to airway obstruction which was an essential factor in patients with dyspnea. Furthermore, we found that pulmonary parenchyma involvement was more common than airway involvement in both severe and critical patients, which may be related to the fact that the H1N1 virus is more likely to involve the alveolar rather than the bronchiolar epithelium.^16^

The current study has some limitations. Firstly, the cohort size was relatively small. Most variables on CT findings showed no significant difference between the two groups after correction for multiple comparisons, so a larger cohort study is needed to consolidate our findings. Secondly, a number of patients with underlying diseases may lead to deviation. Thirdly, several patients had been treated in basic‐level and community hospitals, and early treatment might affect clinical outcomes and imaging presentations. Finally, no lung biopsy was performed, and correlations between CT image features and histopathology could not be obtained.

In conclusion, critical relative to severe group was more likely to present with dyspnea at rest and lower lymphocyte percentage. Chest CT imaging showed more diffuse bilateral involvement and a higher rate of consolidation in critical compared with severe patients. CT findings, along with the help of clinical symptoms and laboratory tests, could contribute to early identification of critical H1N1 pneumonia from those severe patients.

## AUTHOR CONTRIBUTIONS

Mei Yu was responsible for the study design, interpretation of results, and preparation of the first draft of the manuscript. Yuanbo Zhu was responsible for the data analysis. Xiaoyan Qu, Xingyi Hou and Tao Xin were responsible for the data collection. Gangfeng Li was responsible for the editing of manuscript drafts and approval of the final version of the manuscript. All authors read and approved the final version of the publication and gave consent to publication.

## CONFLICT OF INTEREST

The authors declared no potential conflicts of interest with respect to the research, authorship, and publication of this article.

## ETHICS STATEMENT

The study was approved by the ethics committee of our hospital (TDLL‐202205‐01). Informed consent was obtained from all participants included in the study.

## Data Availability

The data that support the results of this study are available from the corresponding author on reasonable request.

## References

[1] Dawood FS , Chung JR , Kim SS , et al. Interim estimates of 2019–20 seasonal influenza vaccine effectiveness—United States, February 2020. MMWR Morb Mortal Wkly Rep. 2020;69(7):177‐182. doi:10.15585/mmwr.mm6907a1 32078591 PMC7043386

[2] Livingston E , Bucher K , Rekito A . Coronavirus disease 2019 and influenza 2019–2020. Jama. 2020;323(12):1122. doi:10.1001/jama.2020.2633 32207769

[3] Hong JL , Li L , Ren MJ , Dai J . Influenza A (H1N1). Radiol Infect Dis. 2015;1:465‐514. doi:10.1007/978-94-017-9882-2_22

[4] Yang P , Deng Y , Pang X , et al. Severe, critical and fatal cases of 2009 H1N1 influenza in China. J Infect. 2010;61(4):277‐283. doi:10.1016/j.jinf.2010.07.010 20670649

[5] Ajlan AM , Quiney B , Nicolaou S , Muller NL . Swine‐origin influenza a (H1N1) viral infection: radiographic and CT findings. AJR am J Roentgenol. 2009;193(6):1494‐1499. doi:10.2214/AJR.09.3625 19933639

[6] Murota M , Johkoh T , Lee KS , et al. Influenza H1N1 virus‐associated pneumonia often resembles rapidly progressive interstitial lung disease seen in collagen vascular diseases and COVID‐19 pneumonia; CT‐pathologic correlation in 24 patients. Eur J Radiol Open. 2020;7:100297. doi:10.1016/j.ejro.2020.100297 33318970 PMC7724381

[7] Schoen K , Horvat N , Guerreiro NFC , de Castro I , de Giassi KS . Spectrum of clinical and radiographic findings in patients with diagnosis of H1N1 and correlation with clinical severity. BMC Infect Dis. 2019;19(1):964. doi:10.1186/s12879-019-4592-0 31718571 PMC6852716

[8] Tanaka N , Emoto T , Suda H , et al. High‐resolution computed tomography findings of influenza virus pneumonia: a comparative study between seasonal and novel (H1N1) influenza virus pneumonia. Jpn J Radiol. 2012;30(2):154‐161. doi:10.1007/s11604-011-0027-6 22180185

[9] Estenssoro E , Rios FG , Apezteguia C , et al. Pandemic 2009 Influenza A in Argentina: a study of 337 patients on mechanical ventilation. Am J Respir Crit Care Med. 2010;182(1):41‐48. doi:10.1164/201001-0037oc 20203241

[10] Sathyamurthy P , Senthil Kumar Dhandapani N . Evaluation of pregnancy, younger age, and old age as independent risk factors for poor hospitalization outcomes in Influenza A (H1N1)pdm09 virus a decade after the pandemic. Cureus. 2020;12(11):e11762. doi:10.7759/cureus.11762 33274169 PMC7707136

[11] Cho WH , Kim YS , Jeon DS , et al. Outcome of pandemic H1N1 pneumonia: clinical and radiological findings for severity assessment. Korean J Intern Med. 2011;26(2):160‐167. doi:10.3904/kjim.2011.26.2.160 21716592 PMC3110848

[12] Cheng Y , Zhao H , Song P , Zhang Z , Chen J , Zhou Y‐H . Dynamic changes of lymphocyte counts in adult patients with severe pandemic H1N1 Influenza A. J Infect Public Health. 2019;12(6):878‐883. doi:10.1016/j.jiph.2019.05.017 31202719 PMC7102863

[13] Chiappini E , Galli L , Azzi A , Resti M , Bonsignori F , de Martino M . Lymphocytopenia as a marker for pandemic influenza a/H1N1 2009 virus infection in children. J Med Virol. 2011;83(1):1‐4. doi:10.1002/jmv.21930 21108332

[14] Coskun O , Avci IY , Sener K , et al. Relative lymphopenia and monocytosis may be considered as a surrogate marker of pandemic Influenza A (H1N1). J Clin Virol. 2010;47(4):388‐389. doi:10.1016/j.jcv.2010.01.007 20133186

[15] Lee HJ , Wanderley M , Rubin V , et al. Lobar pulmonary perfusion quantification with dual‐energy CT angiography: Interlobar variability and relationship with regional clot burden in pulmonary embolism. Eur J Radiol Open. 2022;9:100428. doi:10.1016/j.ejro.2022.100428 35712646 PMC9192795

[16] Valente T , Lassandro F , Marino M , Squillante F , Aliperta M , Muto R . H1N1 pneumonia: our experience in 50 patients with a severe clinical course of novel swine‐origin Influenza A (H1N1) virus (S‐OIV). Radiol Med. 2012;117(2):165‐184. doi:10.1007/s11547-011-0734-1 22020427 PMC7088783

[17] Mauad T , Hajjar LA , Callegari GD , et al. Lung pathology in fatal novel human Influenza A (H1N1) infection. Am J Respir Crit Care Med. 2010;181(1):72‐79. doi:10.1164/rccm.200909-1420OC 19875682

[18] Nakajima N , Sato Y , Katano H , et al. Histopathological and immunohistochemical findings of 20 autopsy cases with 2009 H1N1 virus infection. Mod Pathol. 2012;25(1):1‐13. doi:10.1038/modpathol.2011.125 21874012

[19] Xu Z , Shi L , Wang Y , et al. Pathological findings of COVID‐19 associated with acute respiratory distress syndrome. Lancet Respir Med. 2020;8(4):420‐422. doi:10.1016/s2213-2600(20)30076-x 32085846 PMC7164771

[20] Nin N , Sanchez‐Rodriguez C , Ver LS , et al. Lung histopathological findings in fatal pandemic Influenza A (H1N1). Med Intensiva. 2012;36(1):24‐31. doi:10.1016/j.medin.2011.10.005 22154847

[21] Pantaleão Fontes CA , Dos Santos AASMD , De Oliveira SA , Aidê MA . Influenza A virus H1N1 associated pneumonia—acute and late aspects evaluated with high resolution tomography in hospitalized patients. Respir Med. 2020;15:692‐698. 10.4081/mrm.2020.692 PMC7542991 33117533

